# Supplementary material for: Seminoma and Embryonal Carcinoma Footprints Identified by Analysis of Integrated Genome-Wide Epigenetic and Expression Profiles of Germ Cell Cancer Cell Lines
Source: PLoS One. 2014 Jun 2;9(6):e98330. doi: 10.1371/journal.pone.0098330 (PMC4041891; doi:10.1371/journal.pone.0098330)
Supplement: File S1 — ZIP file containing DMRforPairs output for significant regions. Please start from the html files. (ZIP) [file pone.0098330.s008.zip › figures/5601.pdf]

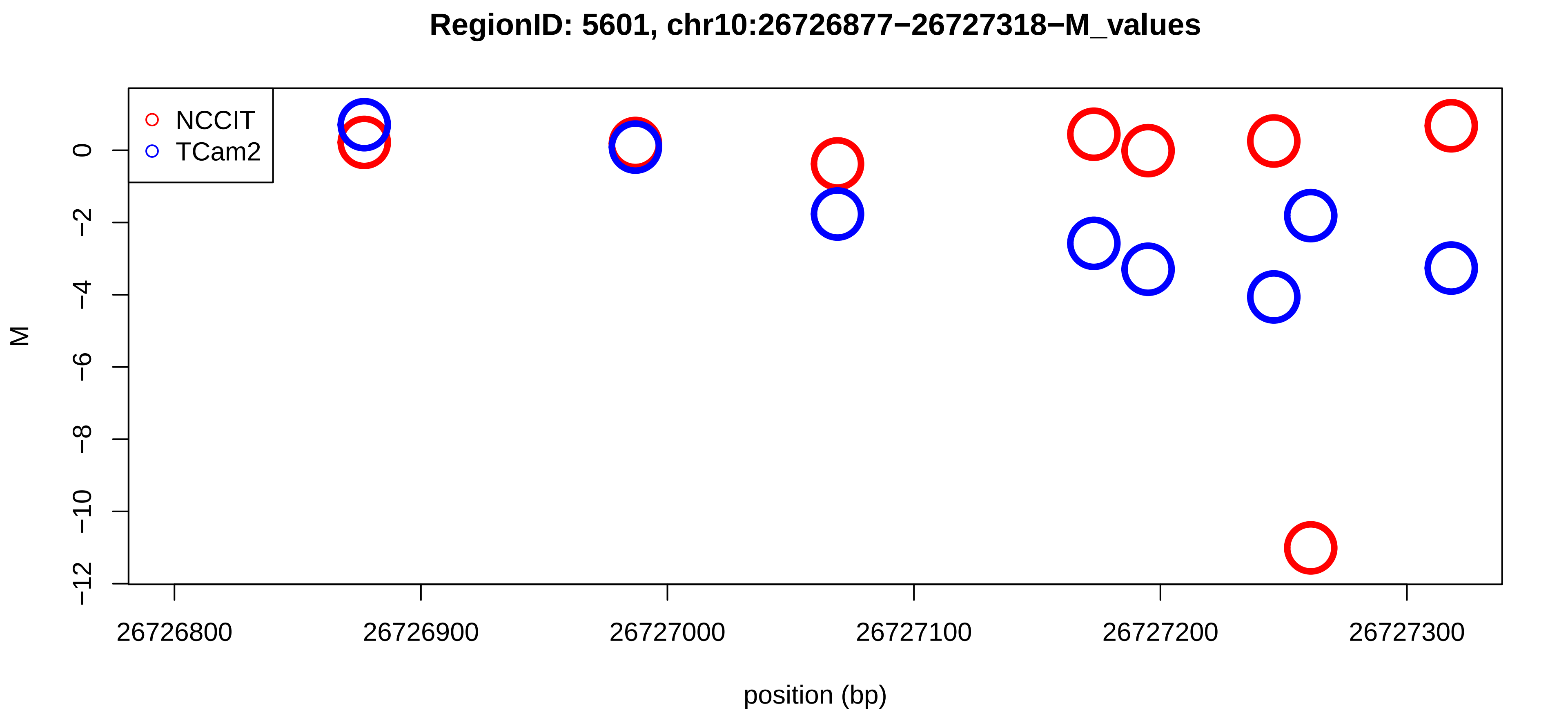
RegionID: 5601, chr10:26726877−26727318−M_values
NCCIT
TCam2
M
0
−2
−4
−6
−8
−10
−12
26726800
26726900
26727000
26727100
26727200
26727300
position (bp)

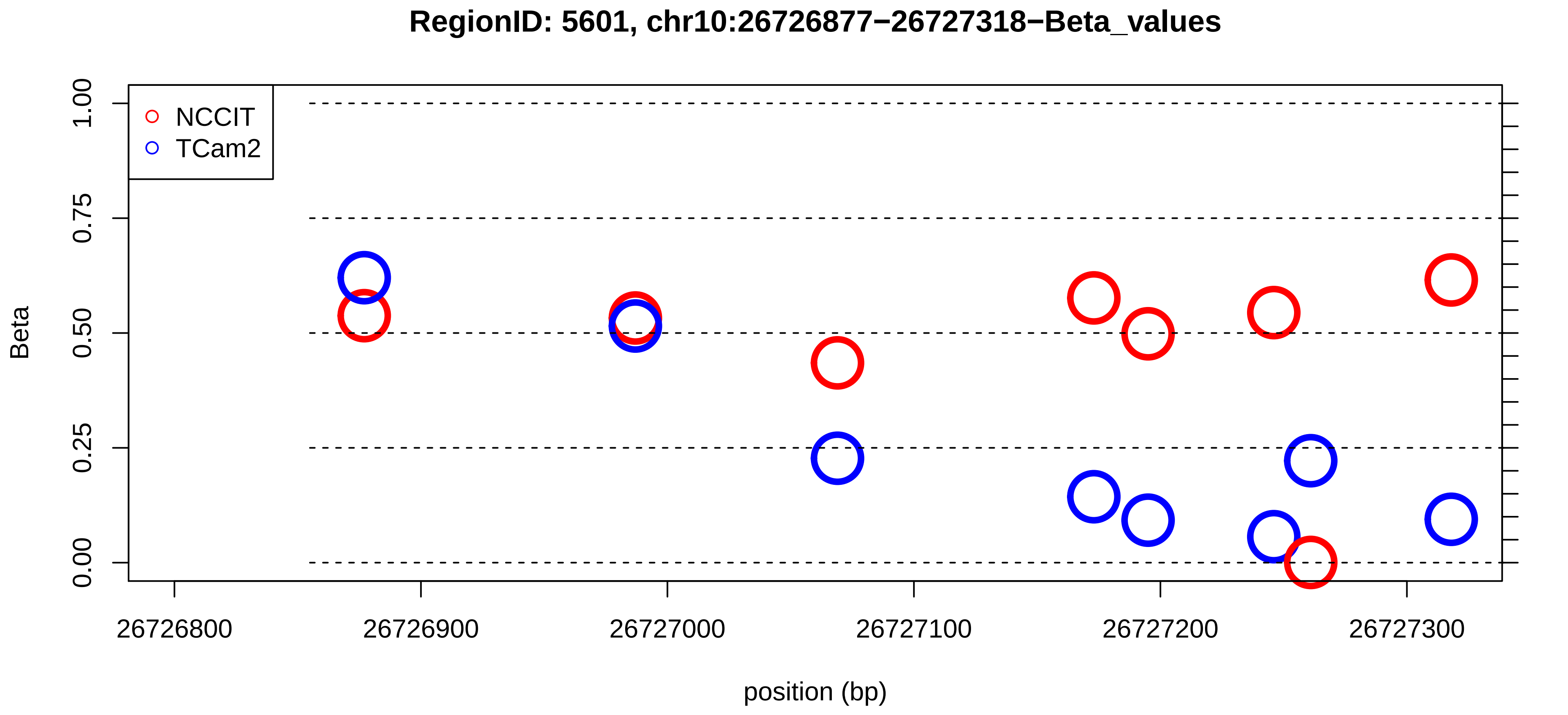
RegionID: 5601, chr10:26726877−26727318−Beta_values
NCCIT
TCam2
Beta
0.00
0.25
0.50
0.75
1.00
26726800
26726900
26727000
26727100
26727200
26727300
position (bp)
